# Some Nervous Effects of Digestive Disorders

**Published:** 1894-01

**Authors:** A. K. Bond

**Affiliations:** Baltimore, Md.


### SOME NERVOUS EFFECTS OF DIGESTIVE DISORDERS.

BY A. K. BOND, M. D., BALTIMORE, MD.

Since the beginning of medicine we have known that soluble poisons taken into the mouth will pass through the walls of the digestive canal into the blood and so produce serious if not fatal disturbance of the general nervous system. And it has been long suspected that, from the contents of the digestive canal itself, poisons are sometimes elaborated which, when absorbed into the blood, produce equally deadly, though perhaps less rapid, disorder of function in the great nerve centers. In recent years clinical observation aided by laboratory research, has not only isolated some of these poisonous digestive products, but traced with considerable accuracy the symptoms they cause. The result encourages us to believe that the source of very many disordered conditions of the blood and nervous systems, hitherto inexplicable, is in the digestive canal.

The condition known as Diabetes Mellitus is believed to be in most cases associated with digestive derangement. I think its nervous phenomena are in some instances directly dependent on imperfect digestion.

I have a diabetic patient, who can eat with impunity at a friend's house amid cheerful company, starchy and even saccharine substances, which at home where digestion was not stimulated by diverting conversation, would produce various nervous phenomena.

Woman is built upon a net-work of nerves, which have to do with their sexual nature, and which lie closest to the surface of her consciousness at a number of points—in the Uterus, Ovaries or Mammary glands, behind the Scapula, in small of the back and along certain regions above or below the Manmae. Any condition which lowers her general health sets these nerves of sex vibrating, even though no local disease can be found in such regions. In any woman whose nerve-force is greatly lowered, the physician may almost certainly detect upon questioning a "spine-ache," "ovary-ache" or a "womb-ache," though there be no local disease in any of these regions, and her sexual life has been normal. In such patients local pains in these sex-nerves are readily called forth by disturbance in other organs, not of the sex-system.

Among such causes, digestive disorder ranks high, and the agent which clears the bowels and restores the intestinal secretion strikes at the roof of the "ache," while one which binds up the bowels, prolongs and establishes the "ache" even though like opium it temporarily deadens pain. A striking illustration occurs in the following case:

I was asked to see a patient under treatment for Cancer or Ulcer of Cervix Uteri. She had within the past week suffered violent paroxysms of pain in lower abdomen, supposed to be due to inflammation of internal sex-organs. For first paroxysm a hypodermic of morphia had been given, which soothed the pain and restored comfort. Next day a recurrence yielded to same treatment. During next few days paroxysms increased to several a day. I first saw patient in a paroxysm, and the position of the feet during the spasm, reminded me of "Hysterical" convulsions and aroused suspicion of nervous irritation instead of pelvic inflammation. After free catharsis by Epsom Salt, there was no return of pain or spasm. The more thoroughly I learn the use of aperient medicines, the less frequently do I have to use opiates. I am convinced that sudden, yet persistent disturbance of the nervous system in its psychic, motor and sensory departments may arise from abnormal conditions in the intestinal tract.

With reference to the influence of abnormal digestion upon the motor nerve-centers, I can cite several cases in which violent convulsions seemed to so originate.

*Case I.* In December, '91, I attended a middle-aged woman for epileptic convulsions, lasting nine hours in spite of chloroform, nitrate of Amyl and enemeta of Pot. Bromid. Drop doses of Croton Oil in Castor Oil brought away a quantity of foul matter from the bowels. In April she had one similar convulsion. Croton Oil again relieved the bowels of masses of very foul tar-like feces.

*Case II.* In June, '93, was called to case of measles in powerful blacksmith of 25 years of age. Rash slight on third day, chilly, nauseated and costive.

Calomel reduced temperature and made him more comfortable. On 9th day the eruption had faded and he was peeling on face. Bowels not moved for two days, restless in sleep, temperature 100°, no urinary disorder. Codea was being taken for chest soreness. Suddenly he waked with delusions and for two days was furiously delirious. Whiskey, Bromide, Chloral, Morphia and Ice were all inaffectual. Magnesii Sulphus. was given, foul passages followed and convalescence set in at once. I had found nothing abnormal about the lungs or other organs.

Dr. Bond also cited other interesting cases and said his desire had been to show that there is a group of disturbances in the sexual, mental, motor and sensory departments of the nervous system, which are due to oft-times obscure, sometimes unsuspected disorders of digestion, and that these often perilous disturbances, though temporarily soothed by opiates require for permanent cure, the cleansing and restoration to normal function of the digestive tract.

